# Cardiac Biomarkers and Their Role in Identifying Increased Risk of Cardiovascular Complications in COVID-19 Patients

**DOI:** 10.3390/diagnostics13152508

**Published:** 2023-07-27

**Authors:** Nagendra Yaluri, Alena Stančáková Yaluri, Pavol Žeňuch, Zuzana Žeňuchová, Štefan Tóth, Peter Kalanin

**Affiliations:** 1 Center of Clinical and Preclinical Research, University Research Park Medipark, P. J. Šafárik University, 040 01 Košice, Slovakia; 2 4th Department of Internal Medicine, P. J. Šafárik University, 040 01 Košice, Slovakia

**Keywords:** cardiovascular disease, biomarkers, SARS-CoV-2 infection, COVID-19

## Abstract

Cardiovascular disease (CVD) is a global health concern, causing significant morbidity and mortality. Both lifestyle and genetics influence the development of CVD. It is often diagnosed late, when the treatment options are limited. Early diagnosis of CVD with help of biomarkers is necessary to prevent adverse outcomes. SARS-CoV-2 infection can cause cardiovascular complications even in patients with no prior history of CVD. This review highlights cardiovascular biomarkers, including novel ones, and their applications as diagnostic and prognostic markers of cardiovascular complications related to SARS-CoV-2 infection. Patients with severe SARS-CoV-2 infection were shown to have elevated levels of cardiac biomarkers, namely N-terminal pro-brain natriuretic peptide (NT-pro-BNP), creatine kinase-myocardial band (CK-MB), and troponins, indicating acute myocardial damage. These biomarkers were also associated with higher mortality rates and therefore should be used throughout COVID-19 patient care to identify high-risk patients promptly to optimize their outcomes. Additionally, microRNAs (miRNAs) are also considered as potential biomarkers and predictors of cardiac and vascular damage in SARS-CoV-2 infection. Identifying molecular pathways contributing to cardiovascular manifestations in COVID-19 is essential for development of early biomarkers, identification of new therapeutic targets, and better prediction and management of cardiovascular outcomes.

## 1. Introduction

Cardiovascular disease (CVD) is a heterogeneous group of diseases affecting the heart and blood vessels. It is the leading cause of mortality worldwide, accounting for over 17 million deaths per year [1]. CVD includes coronary artery disease, heart failure, valvular heart disease, arrhythmias, and stroke [2]. Risk factors for CVD include high blood pressure, high cholesterol, smoking, obesity, diabetes, and a family history of the disease [2]. Prevention and management of CVD requires lifestyle changes, such as a healthy diet, regular physical activity, smoking cessation, and stress management, as well as medical interventions, such as medications, surgery, and other procedures (American Heart Association, 2022) [3]. Early detection and treatment of CVD can significantly improve outcomes and reduce the risk of complications [2].

Biomarkers are measurable indicators that can be used to diagnose or monitor the progression of a disease or the effects of a treatment [4]. In the context of cardiovascular disease, biomarkers are substances or molecules that can be measured in the blood, urine, or other bodily fluids and are associated with the presence or severity of CVD [5].

Studies have reported associations of SARS-CoV2 infection with various cardiovascular complications, such as myocarditis, heart failure, and arrhythmias. On the other hand, individuals with pre-existing cardiovascular diseases, such as hypertension, coronary artery disease, and heart failure, were found to have a higher risk of severe COVID-19 illness and higher mortality [6,7]. In some cases, these complications may occur in patients with no prior history of cardiovascular disease [8,9].

Cardiovascular biomarkers can include a wide range of substances, such as proteins, enzymes, lipids, and hormones, that are indicative of various aspects of cardiovascular function or dysfunction [5]. The most commonly studied biomarkers include creatine kinase-myocardial band (CK-MB), troponin, N-terminal pro-brain natriuretic peptide (NT-proBNP), and D-dimer. Elevated levels of cardiac troponin have been observed in COVID-19 patients with cardiac injury, indicating myocardial damage [10,11]. Elevated levels of CK have also been observed in COVID-19 patients with cardiac injury [12]. BNP and NT-proBNP biomarkers are associated with cardiac stress and have been found to be elevated in COVID-19 patients with cardiac injury [13,14]. Elevated levels of D-dimer, a biomarker of coagulation activation, have been observed in COVID-19 patients with thromboembolic events [15]. Other biomarkers that have been associated with COVID-19 and cardiovascular complications include interleukin-6 (IL-6), C-reactive protein (CRP), and ferritin, which are markers of inflammation and have been linked to a hyperinflammatory response in severe COVID-19 cases [16,17]. It is important to note that the interpretation of cardiac biomarkers in COVID-19 patients should be made in the context of the patient’s clinical status and other relevant factors, and not solely rely on the biomarker values.

Along with traditional cardiovascular biomarkers, microRNAs (miRNAs) have been proposed as a powerful class of biomarkers for COVID-19.

Almost 60% of the human genome is transcribed into noncoding RNAs, with microRNAs being the most studied class of noncoding RNAs [18,19]. MicroRNAs are short, single-stranded RNA molecules which regulate gene expression by binding to complementary mRNAs, leading to mRNA degradation or translational inhibition [20,21,22]. MicroRNAs were found to regulate the expression of over 60% of protein-coding genes in mammals, including those involved in the antiviral response [23]. Specifically, cellular microRNAs can bind to RNA virus genomes, exerting an antiviral effect [20,24,25]. In the case of COVID-19, microRNAs targeting specific genes, such as the S, M, N, E, and open reading frame 1ab genes, may limit SARS-CoV-2 invasion and replication, suppressing the virus’s ability to infect cells [23]. Additionally, miRNAs play a key role in regulating inflammation-related mediators and immune responses, including those involved in viral-mediated inflammation, such as COVID-19 [23,26].

Moreover, miRNAs have been implicated in regulating COVID-19 complications, particularly cardiovascular (CV) events, given their potential role as causal factors in disease progression [27]. MiRNAs can influence susceptibility to SARS-CoV-2 infection by regulating immune responses and inflammation, and also directly by modulating cell damage caused by the virus or by cytokine storm [23]. MiRNAs dysregulation has also been associated with a variety of CV alterations, making them potentially useful as biomarkers and prognostic predictors in COVID-19 patients [23]. Further research on the role of miRNAs in COVID-19 and its cardiovascular complications is warranted.

**Figure 1:**
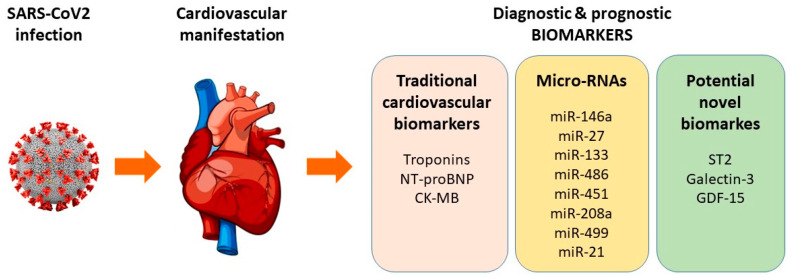
Established and novel biomarkers and micro-RNAs as biomarkers of cardiovascular manifestations in COVID-19.

The aim of this review is to evaluate some of the biomarkers of cardiovascular complications in COVID-19 patients (Figure 1), which can help to optimize their outcomes.

## 2. Traditional Cardiovascular Biomarkers

### 2.1. Troponin

Several previous coronaviruses have been associated with cardiac complications such as acute-onset heart failure, arrhythmias, cardiac arrest, sub-clinical diastolic impairment, and cardiomegaly. Troponin-T (TnT) has been suggested as a possible prognostic biomarker for COVID-19 patients in numerous clinical studies, with elevated levels being linked to myocardial injury and increased mortality rates [12,28,29,30]. Elevated high sensitivity TnT (hs-TnT) levels in COVID-19 patients were associated with an increased risk of negative outcomes, including ARDS, mechanical ventilation, and ICU admission. About 36% of COVID-19 patients had elevated hs-TnT levels, making it a potentially useful biomarker for identifying high-risk patients. The exact mechanism behind hs-TnT elevation in COVID-19 patients is not fully understood, but it may be due to direct heart cell damage caused by the virus or systemic inflammation and cytokine release [28].

The association between cardiac injury and mortality in COVID-19 patients was investigated in 416 hospitalized patients with confirmed COVID-19 in the study of Shi et al. [12]. The cardiac status of patients was evaluated through blood tests, ECG, and echocardiography. The study found that 19.7% of COVID-19 patients had cardiac injury, as indicated by an increase in cardiac troponin I (cTnI) levels above the 99th percentile upper reference limit. The mortality rate was significantly higher in COVID-19 patients with cardiac injury (51.2%) compared to patients without cardiac injury (4.5%). The study also reported an association of older age, preexisting cardiovascular disease, and higher SOFA scores with cardiac injury. However, the study had limitations, including a small sample size and potential confounding variables. Further research is necessary to confirm these findings and explore the underlying mechanisms of cardiac injury in COVID-19 patients [12]. These results indicate that myocardial injury is a common complication in hospitalized COVID-19 patients and is associated with a significantly higher risk of mortality [12,29]. Similar results were obtained in another study which included 187 patients who died from COVID-19, 25.1% of whom had a history of cardiovascular disease. Similarly, the patients who died from COVID-19 had higher levels of cardiac biomarkers, including cTnI and CK-MB, indicating myocardial injury [30].

A multicenter study including 543 patients with COVID-19 showed that patients with elevated hs-cTnT levels were more likely to experience fever and respiratory symptoms, had a higher prevalence of cardiovascular disease and hypertension, and were more likely to be admitted to the intensive care unit [31]. These findings suggest that monitoring hs-cTnT levels in COVID-19 patients could help detect cardiac involvement early and identify individuals at risk of cardiac complications.

It is clear from the above literature that COVID-19 is associated with increased rates of myocardial injury, and elevated TnT levels are a reliable marker for myocardial damage and a negative prognostic factor for COVID-19 patients. Early identification and monitoring of TnT levels in COVID-19 patients, particularly those with a previous history of heart disease, could help to stratify patient risk and inform treatment decisions. However, further research is needed to establish the exact mechanisms behind the cardiac complications seen in COVID-19 patients and to develop effective treatments to mitigate these complications. Additional research is necessary to ascertain the precise mechanisms underlying the cardiac complications observed in individuals with COVID-19 and to formulate efficacious interventions for managing these complications.

### 2.2. B-Type Natriuretic Peptide

NT-proBNP is a cardiac biomarker released by the cardiac ventricles in response to increased wall tension and stretching, reflecting heart function in heart failure [32,33]. Its levels in the blood are directly related to the severity of heart failure, making it a useful biomarker for diagnosis, management, and prognosis of heart failure [8,34]. NT-proBNP exerts its effects on the cardiovascular system via the natriuretic peptide receptor type A (NPR-A), upon binding to which the cyclic guanosine monophosphate (cGMP) signaling pathway is activated, resulting in vasodilation, natriuresis, and diuresis, leading to reduced blood pressure and fluid overload [8,35]. However, chronic activation of the NT-proBNP/NPR-A/cGMP pathway in response to sustained cardiac stress can lead to adverse effects on the heart and blood vessels, such as myocardial fibrosis, hypertrophy, and vascular remodeling [35,36].

Several studies have linked cardiac biomarkers, including NT-proBNP, with higher mortality risk in COVID-19 patients. Chen et al. found that several cardiac biomarkers, including hs-cTnI, NT-proBNP, and myoglobin, were significantly elevated in deceased COVID-19 patients compared to survivors [37].

Caro-Codón et al. found that NT-proBNP levels were elevated in COVID-19 patients compared to healthy controls and that higher levels were associated with more severe disease, a higher risk of mortality, and a longer hospital stay. Additionally, they found that NT-proBNP levels decreased during hospitalization in survivors, but remained elevated or continued to increase in non-survivors. The study suggests that NT-proBNP may be a useful biomarker for predicting disease severity and outcomes in COVID-19 patients [38].

Additionally, Gao et al. reported that patients with elevated NT-proBNP levels on admission had a significantly higher risk of mortality, longer hospital stays, and greater need for mechanical ventilation [39]. Inciardi et al. found that patients with pre-existing cardiac disease, who had higher levels of cardiac biomarkers, such as troponin and NT-proBNP, were more likely to experience in-hospital mortality [40].

The Cardio-COVID-Italy Multicenter Study [41] assessing the combined prognostic value of natriuretic peptides (NPs) and troponin in 341 hospitalized COVID-19 patients showed that patients with elevated levels of both NPs and troponin had a higher risk of death, even after adjusting for various factors. Furthermore, NPs provided risk stratification, identifying patients with a worse prognosis even in the presence of normal troponin values. These findings underscore the importance of both NPs and troponin as prognostic markers in COVID-19.

These studies suggest that NT-proBNP may be a useful prognostic biomarker in severe COVID-19 patients, allowing for earlier identification of those at higher risk of poor outcomes and potentially guiding treatment decisions. More research is needed to validate these findings and elucidate the potential mechanisms underlying the relationship between NT-proBNP and COVID-19 outcomes. Further studies are necessary to confirm these observations and explore the intricate pathways through which NT-proBNP may influence the course of COVID-19.

### 2.3. Creatine Kinase-Myocardial Band

The term “creatine kinase myocardial band” refers to the isoenzyme CK-MB, which is primarily found in the heart muscle and is released into the bloodstream when there is damage or injury to the heart muscle, such as in a myocardial infarction [42]. CK-MB is one of several biomarkers that can be used to diagnose myocardial infarction, and its release into the bloodstream is believed to occur within 3–12 h of the onset of myocardial infarction and typically peaks within 24 h. The level of CK-MB in the blood can be used to estimate the size of the infarct, with higher levels indicating a larger infarct [42]. However, the use of CK-MB has declined in recent years with the development of more sensitive biomarkers such as cardiac troponins [42].

Several studies have shown that CK-MB levels in COVID-19 patients may be useful in identifying those at higher risk of mortality. The study by Ji et al. found that patients with elevated levels of CK-MB, myoglobin, and high-sensitivity cardiac troponin I (hs-cTnI) had a significantly higher risk of developing acute cardiac injury compared to those with normal levels of these markers [43]. Lippi et al. reported that several studies have shown elevated levels of CK-MB in COVID-19 patients, indicating cardiac damage. They further suggested that hs-TnI and CK-MB levels could serve as important biomarkers for identifying COVID-19 patients with cardiac damage [44]. Sha et al. found that the levels of CK and CK-MB were significantly higher in patients with severe COVID-19 disease compared to those with non-severe disease, and the levels of CK and CK-MB correlated positively with the severity of the disease [45]. The study by Shi et al. found that patients with cardiac injury had higher levels of troponin I, myoglobin, and CK-MB than those without cardiac injury. This and other studies also provide evidence for the use of cardiac biomarkers, such as troponin I, myoglobin, and CK-MB, in the diagnosis and prognosis of cardiac injury in COVID-19 patients [12,46]. Yang et al. found that elevated CK-MB levels were associated with an increased risk of mortality in COVID-19 patients [47].

It is worth noting that while these studies mention CK-MB, they do not all focus specifically on its role in COVID-19. Instead, they examine the association of cardiac markers, including CK-MB, with COVID-19-related complications such as acute cardiac injury and mortality.

Overall, the above studies provide important insights into the abnormal changes in myocardial enzymes in COVID-19 patients and highlight the potential role of CK and CK-MB in the diagnosis and prognosis of COVID-19-induced myocardial injury. This information may be helpful for clinicians in identifying patients who may require more aggressive monitoring and treatment to prevent adverse cardiac events. Additional investigation is required to gain a comprehensive understanding of the mechanisms involved in cardiac injury during COVID-19 and to determine the significance of cardiac markers like CK-MB in prognosticating outcomes for individuals affected by the disease.

## 3. MicroRNAs (miRNAs) as Potential Biomarkers in COVID-19-Associated Cardiovascular Complications

MiRNAs are small non-coding RNAs with important roles in the regulation of gene expression [48,49]. MiRNAs are transcribed from genomic DNA and undergo a series of processing steps to become mature miRNAs that can interact with target mRNAs [49]. Dysregulation of miRNAs has been implicated in many diseases, including cancer, CVD, and neurological disorders [49]. MiRNAs have been investigated as potential biomarkers for various diseases. For example, in cancer, overexpression of miR-21 was detected in colorectal cancer tissue compared to adjacent normal tissue, and the expression level correlated with the stage and prognosis of the disease [50]. In CVD, miRNAs have also emerged as promising biomarkers, particularly for the diagnosis and prognosis of acute myocardial infarction (AMI) and heart failure [51].

Circulating levels of miR-208a were significantly elevated in patients with AMI compared to healthy controls, and its levels correlated with the extent of myocardial injury [52]. MiR-1, miR-133a, and miR-208b were also significantly elevated in patients with AMI, and a combination of these miRNAs had high sensitivity and specificity for the diagnosis of AMI [53]. For heart failure, circulating levels of miR-423-5p were significantly elevated and correlated with disease severity and outcomes, while a panel of six miRNAs was able to predict mortality and hospitalization in patients with heart failure [54,55]. MiRNAs are attractive biomarkers for CVD due to their stability in blood and other bodily fluids, tissue-specific expression patterns, and involvement in various aspects of CVD pathophysiology [51]. However, further research is needed to validate these findings and to develop standardized methods for miRNA detection and analysis.

Recent research has suggested that miRNAs may also be involved in the cardiovascular complications of COVID-19 [12]. Studies have proposed that miRNAs may contribute to the development of COVID-19-related cardiovascular pathology by regulating the expression of genes involved in inflammation, oxidative stress, and endothelial dysfunction [56]. Moreover, dysregulation of miRNAs could contribute to the development of COVID-19-associated coagulopathy, a common complication of the disease that can lead to thrombosis and other cardiovascular problems [57].

Although the research in this field is still limited, investigating miRNAs involved in both SARS-CoV-2 infection and cardiovascular pathology can improve our understanding of the relationship between COVID-19 and increased cardiovascular risk.

### 3.1. MiR-146a

Sabbatinelli et al. [58] investigated levels of various miRNAs, including miR-146a-5p, in COVID-19 patients treated with tocilizumab (TCZ), an inhibitor of IL-6 signaling. Previous studies have shown a correlation between IL-6 levels and COVID-19 severity [59]. MiR-146a-5p negatively regulates the NF-κB transcription factor, which controls the gene encoding IL-6 [60]. Sabbatinelli et al. [58] discovered a contrasting association between miR-146a and IL-6 in COVID-19 patients, with higher levels of IL-6 but lower levels of miR-146a-5p in COVID-19 patients than in healthy controls. Furthermore, the levels of miR-146a were higher in COVID-19 patients who responded to TCZ than in non-responders, in whom the lowest miR-146a-5p levels were associated with more severe COVID-19 symptoms. These findings suggest that altered serum levels of miR-146a could serve as a valuable marker of the clinical response to COVID-19 treatment.

Reduced levels of circulating miR-146a, found in patients with obesity, diabetes and hypertension, could potentially elucidate the heightened severity of COVID-19 cases in such patients [61]. It is also plausible that COVID-19 patients with lower miR-146a expression levels have higher levels of IL-6 and other pro-inflammatory cytokines, increasing the risk of cytokine storms. The role of IL-6 in COVID-19 progression was also highlighted by work by Vasuri et al. [62] reporting higher levels of IL-6 in COVID-19 patients than in healthy controls. Gao et al. [63] demonstrated that miR-146a attenuates sepsis-induced cardiac dysfunction. Another study by Oh et al. [64] found upregulated miR-146a expression in failing cardiomyocytes, and its overexpression suppressed small ubiquitin-like modifier 1 (SUMO1) expression, leading to cardiac contractile dysfunction by reducing sarco-endoplasmic reticulum calcium ATPase-2 (SERCA-2) sumoylation. Conversely, Huang et al. [65] showed elevated miR-146a expression levels in peripheral blood, which correlated with IL-6 and TNF-α expression, as well as plaque vulnerability and the degree of stenosis in carotid atherosclerosis. In another study, miR-146a has been found to have a cardioprotective function, promoting cardiomyocyte viability and protecting against oxidative stress [66]. These findings suggest that miR-146a expression may exhibit opposite patterns in CVD and COVID-19.

Altogether, there seems to be a strong connection between miR-146a and hyper-inflammatory processes observed in both SARS-CoV-2 infection and atherosclerosis. Imbalance between miR-146a and IL-6 may cause an impairment of the regulation of pro-inflammatory cytokines, leading to their excessive secretion and a hyper-inflammatory state. Downregulation of miR-146a may result in an insufficient host antiviral response, cytokine storm, and a lack of feedback mechanisms to limit inflammatory tissue damage [67].

### 3.2. MiR-27

Studies have demonstrated that the miR-27 levels are altered in individuals with cardiac remodeling, atherosclerosis, and coronary artery disease [68,69]. MiR-27 was associated with cardiac remodeling and atherosclerotic plaque formation by its effects on macrophages involved in tissue fibrosis and by the regulation of cholesterol synthesis [66]. Upregulation of miR-27a was also suggested to play a role in promoting angiogenesis during ischemia-reperfusion [70].

MiR-27 was found to be downregulated in individuals affected by SARS-CoV-2 infection [62,71], and it has been proposed to function as a suppressor of IL-6 expression. In a study by Vasuri et al. [62], a significant decrease in miR-27a-5p levels was observed in atherosclerotic and normal femoral arteries from a COVID-19 patient with pneumonia, bilateral interstitial lesions, and cardiovascular comorbidities. Also, IL-6 mRNA and protein expression was increased in this patient, indicating a hyperinflammatory state. The study proposed a link between perivascular endothelialitis and miR-27a-5p, and emphasized the association of IL-6 expression with miR-27a-5p in SARS-CoV-2 pathology.

### 3.3. MiR-133

Gutmann et al. [72] examined the association between circulating miRNAs and the severity of COVID-19 as well as mortality during 28-day hospitalization in the intensive care unit (ICU). The study reported differential expression of 60 miRNAs, including miRNAs derived from cardiomyocytes, platelets, endothelial cells, and hepatocytes, depending on the severity of the disease. Notably, two specific miRNAs, miR-133a (a myocyte-derived miRNA also known as MyomiR) [73] and miR-208b (derived from cardiomyocytes) [74], were detected in patients with severe disease. MiR-133a, reflecting an inflammation-induced damage to myocytes, was found to be associated with 28-day mortality and exhibited a negative correlation with neutrophil counts and markers of neutrophil degranulation, namely myeloperoxidase [72]. Critically ill patients with cardiopulmonary diseases demonstrated the highest levels of miR-133a. MiR-133a is known to play a significant role in essential processes related to cardiac hypertrophy, cardiac fibrosis, and arrhythmia, such as the proliferation, hypertrophic growth, differentiation, and electrical conduction of cardiac cells and their survival [75]. Additionally, conditions like myocardial injury [76] and chronic obstructive pulmonary disease (COPD) [77] have been known to elevate circulating levels of miR-133a. The rise in circulating miR-133a is likely attributed to neutrophil degranulation and extravasation, leading to myocyte damage [78]. Moreover, there is evidence suggesting that neutrophils may serve as a secondary source of circulating miR-133a [79].

### 3.4. MiR-486

MiR-486, expressed in the heart and muscle tissues, regulates various protein networks related to cardiomyocyte survival, myogenesis, myotube survival/differentiation, and cardiac progenitor cell proliferation. It negatively regulates PIM1 kinase, an essential component of the cyclin in cardiac progenitor cells, thereby contributing to myocardial homeostasis [80]. MiR-486-5p promotes cell death by increasing the accumulation of superoxide anions and causing DNA damage, and is associated with decreased cell proliferation. These mechanisms are closely linked to heart failure and cardiac remodeling. MiR-486 was also found to influence the activation and differentiation of T- and B-lymphocytes, and to downregulate the expression of the anti-inflammatory cytokine IL-10, thereby exhibiting a pro-inflammatory role [71]. Reduced expression levels of miR-486-5p have been proposed to contribute to immune system impairment and increased disease severity by facilitating viral replication [71]. Notably, miR-486-5p ranks among the most prominently downregulated miRNAs in lung tumor tissues and plays a significant role in the advancement of lung cancer [81]. The role of miR-486-5p in COVID-19 is uncertain. It was found to be downregulated in COVID-19 patients but upregulated in patients with atrial fibrillation, suggesting that COVID-19 may not impact arrhythmias via miR-486-5p. However, Li et al. [82] discovered that miR-486-5p is overexpressed in arrhythmia patients and associated with suppressing the sinoatrial node’s function.

Elevated miR-486-5p levels in arrhythmia patients may indicate prolonged inflammatory responses and increased susceptibility to severe COVID-19 states with hyper-inflammatory conditions or cytokine storms. Additionally, studies have shown significant downregulation of miR-486 in the heart after cardiac ischemia/reperfusion injury [83].

### 3.5. MiR-451

MiR-451 exhibits expression across multiple systems, including the urinary, respiratory, and digestive systems. It assumes a regulatory function in diverse physiological and pathological processes, such as hematopoiesis, epithelial cell polarity, and embryogenesis [84]. MiR-451 is upregulated in atrial fibrillation patients, protecting against cell death caused by ischemia-reperfusion injury through the cyclooxygenase-2 pathway, associated with arrhythmias [85]. While the precise mechanism of miR-451 in atrial fibrillation remains unclear, it can be seen as an adaptive response. MiR-451 downregulation in COVID-19 patients is linked to severe outcomes, potentially promoting the expression of pro-inflammatory cytokines and viral replication [69]. Reduced miR-451 levels compromise apoptotic protection, increasing susceptibility to CVD and contributing to tissue damage, fibrosis, and cardiac remodeling [86]. MiR-451a is recognized as a suppressor of IL-6R translation. Yang et al. [87] observed a notable increase in mRNA expression of IL-6R and downregulation of miR-451a in COVID-19 patients. This dysregulation may lead to increased expression of IL-6 protein, which has implications for the inflammatory response associated with COVID-19.

### 3.6. Other MiRNAs

In a study conducted by Garg et al. [69], comparing the levels of several cardiac-specific circulating miRNAs among COVID-19 patients and individuals with influenza-associated acute respiratory distress syndrome (Influenza-ARDS), it was shown that COVID-19 patients exhibited significantly increased serum concentrations of miR-155 (linked to inflammation), miR-208a and miR-499 (related to myocardial/cardiomyocyte damage), and miR-21 (associated with cardiac fibroblast and endothelial cell dysfunction) compared to healthy controls. Interestingly, the altered levels of miR-155 and miR-499 could differentiate between COVID-19 and influenza-ARDS patients, despite the phenotypic similarities of the two diseases. This suggests a specific cardiac response and involvement in COVID-19. MiR-21 is known to modulate the ERK-MAP kinase signaling pathway in cardiac fibroblasts, upregulation of which contributes to interstitial fibrosis and cardiac dysfunction, particularly in heart failure [88]. Elevated levels of miR-155 in COVID-19 patients have been correlated with cardiovascular damage, fibroblast proliferation, endothelial inflammation, apoptosis promotion, and cardiomyocyte pyroptosis. These changes lead to hypertrophy, ventricular dysfunction, and ultimately heart failure. Furthermore, increased expression of miR-155 has been observed in severe COVID-19 patients, which is associated with a higher incidence of arrhythmias, indicating its potential as a marker for heart damage [89].

### 3.7. MiRNAs in Relation to Established Cardiac Biomarkers

Several miRNAs have been correlated with established cardiac biomarkers in small-scale studies; however, it is difficult to validate them in large cohorts of patients, partly because of the lack of standardized assays for the detection of miRNAs. For example, the levels of miR-208a-3p and miR-499-5p found to be elevated in the plasma of viral-cardiomyopathy patients correlated positively with myocardial damage assessed by troponin T levels [90]. Similarly, in a study of 424 patients with acute coronary syndrome, miR-208b and miR-499 showed higher expression levels in myocardial infarction (MI) patients compared with non-MI patients, and they were well correlated with cardiac troponins, although their diagnostic value was not superior [91]. Furthermore, increase in miR-1, miR-133a, and cardiac-enriched miR-208a levels in the first 4 h of cardiac injury correlated with cTnT levels [92]. Nevertheless, a study examining several miRNAs (miR-133a, miR-208b, miR-223, miR-320a, miR-451, and miR-499) in 1155 patients with acute chest pain demonstrated that none of the tested miRNAs outperformed cTnT in its diagnostic and prognostic accuracy [93].

The above studies indicate that, in future, miRNAs can be used as prognostic markers, and may have therapeutic potential for assessing COVID-19 severity and its related cardiovascular complications and to develop effective miRNA-based therapies. Nanoparticle-based delivery systems may offer a promising approach for delivering miRNA therapeutics to target cells. However, additional research with the help of computational meta-analysis and bioinformatic tools is necessary to validate these findings and determine the clinical applicability of miRNAs as prognostic markers and to assess their therapeutic potential in COVID-19 patients.

## 4. Potential Novel Biomarkers

### 4.1. ST2

ST2 is a biomarker belonging to the interleukin 1 (IL-1) receptor family, which plays a role in cell proliferation [94,95]. It is expressed in various cell types, including macrophages, neutrophils, lymphocytes, endothelial cells, cardiomyocytes, osteoclasts, osteoblasts, and adipocytes. It is found in two biologically relevant isoforms: the transmembrane form ST2L and the soluble secreted form sST2 [96,97].

ST2L acts as a receptor for interleukin 33 (IL-33). IL-33 can be secreted by various cell types as a response to damage. The interaction between IL-33 and ST2L leads to activation of macrophages, mast cells, and Th2 cells, and to secretion of cytokines and chemokines that are involved in the immune response [98,99,100]. It has also been described that ST2L/Il-33 interaction exerts cardioprotective effects by reducing cardiac fibrosis, hypertrophy, and apoptosis [101,102].

On the other hand, sST2 acts as a decoy receptor for IL-33, preventing its interaction with ST2L and thus inhibiting the protective effects of IL-33/ST2L signaling [103].

In recent years, sST2 has gained attention as a diagnostic and prognostic biomarker for cardiovascular diseases including heart failure and acute and chronic myocardial infarction, but also for pulmonary diseases like asthma and acute respiratory distress syndrome (ARDS), sepsis, and gastrointestinal diseases [104,105]. It has also been suggested as a potential tool for managing COVID-19 patients.

In a study by Zeng et al. involving patients with COVID-19, it was found that severe cases had significantly higher levels of sST2 compared to mild cases and healthy controls. Furthermore, elevated sST2 levels were associated with short-term mortality and correlated with other markers of inflammation such as CRP and procalcitonin [106]. In another study, COVID-19 patients with sST2 levels above 58.9 ng/L had a higher risk of ICU admission or death, and peak concentrations of sST2 were reached 48–72 h after admission [107]. Elevated levels of sST2 were also observed in ARDS patients [108].

This increase in sST2 concentration in the blood may impair the protective roles of the IL-33/ST2L system in the heart, leading to myocardial remodeling and potentially worse outcomes in COVID-19 patients [105,109]. Similarly, inflammatory mediators IL-6 and TNFα also contribute to myocardial remodeling and worse outcomes in COVID-19 [110].

The results of the above studies suggest that monitoring serum sST2 levels could be beneficial for early screening of inflammatory status and critical illness in COVID-19 patients. Further studies are needed to explore the potential use of sST2 as a prognostic biomarker of cardiovascular complications in COVID-19 patients. This includes examining the circulating levels of sST2 in the blood and investigating the expression of IL-33 and ST2 genes in the heart of COVID-19 patients.

### 4.2. Galectin-3

Galectin-3 is a β-galactoside-binding lectin that plays a role in various biological processes in various organs, including cell proliferation, apoptotic regulation, inflammation, fibrosis, and host defense [111]. It is found in various organs, including the heart, lungs, and kidneys, and is primarily secreted by macrophages and fibroblasts in response to acute inflammatory processes [111,112,113,114]. It has also been described to be involved in the pathogenesis of atherosclerosis and cardiovascular disease [14,15,115,116], and has been recommended as a biomarker for heart failure [16,17] and for assessing the risk in patients with acute cardiovascular disease [112,117,118]. For example, a study by Chen et al. described a crucial role of galectin-3 in platelet activation and thrombus formation in patients with coronary artery disease [119] with proposed therapeutic potential [120].

Due to the involvement of galectin-3 in inflammatory processes, recent studies have also examined galectin-3 as a biomarker of severity of COVID-19. A study of COVID-19 patients hospitalized with acute respiratory failure reported that those who experienced fatal events within a 30-day follow-up period had higher levels of galectin-3. Galectin-3 was a significant predictor of mortality even after considering other biomarkers such as IL-6 and CRP, and elevated galectin-3 levels were associated with ICU admission and a higher risk of ARDS [121]. Similarly, another study demonstrated significantly higher galectin-3 levels in patients with severe COVID-19 compared to non-severe cases or healthy individuals [122]. The latest study, including 280 COVID-19 patients classified into four different severity groups, revealed significant differences in galectin-3 levels among patients of varying COVID-19 severity, with the highest levels of galectin-3 but also IL-1β, TNF-α, IL-12, and IL-10 in the critical (highest severity) [123] group.

COVID-19 is also associated with higher thrombotic and thromboembolic risk [124,125]. According to the study by Puccini et al. [22], galectin-3 can also be considered as a marker for increased hypercoagulability in COVID-19 since its levels correlated positively with platelet and coagulation markers of thrombogenicity in COVID-positive patients. However, the exact involvement of galectin-3 in the pathophysiology of thrombotic disease in COVID-19 is yet unknown.

### 4.3. GDF-15

Growth differentiation factor 15 (GDF-15) is an inflammatory biomarker belonging to the transforming growth factor ß superfamily. It is widely distributed in various organs, where it is secreted by macrophages, endothelial cells, and cardiomyocytes during oxidative stress, tissue injury, or inflammation [126,127]. Although the full effects of GDF-15 are not yet fully understood, it has shown potential cardioprotective effects. For example, GDF-15 has been established as a robust biomarker of heart failure in cases with both preserved and reduced ejection fraction [126,128,129]. Additionally, elevated levels of GDF-15 have been associated with increased morbidity and mortality in patients with inflammatory diseases [130,131,132].

GDF-15 has also been shown as a significant predictor for important clinical outcomes in COVID-19. A study conducted in Norway [131] in hospitalized COVID-19 patients found significantly higher levels of GDF-15 levels in those who required ICU admission or experienced death during hospitalization. This association remained significant even after considering other relevant biomarkers and clinical comorbidities. The study also identified correlations between GDF-15, detectable SARS-CoV-2 viremia, and hypoxemia. Another study [133] observed increased levels of GDF-15 in patients with fatal outcomes of COVID-19, and the association remained significant even after adjusting for sepsis-related organ failure assessment. Smaller studies have also reported associations between GDF-15 and COVID-19 severity, with dynamic changes in GDF-15 closely linked to disease progression [133,134,135,136]. Moreover, elevated GDF-15 levels were observed in COVID-19-related ARDS cases treated in the ICU, particularly among non-survivors [137,138].

In summary, GDF-15 serves as an important biomarker in COVID-19, providing insights into disease severity and predicting clinical outcomes. Further studies are needed to examine its potential as a biomarker of cardiovascular complications of COVID-19.

## 5. Conclusions

Cardiac biomarkers are substances that are measured in the blood and can help clinicians predict an individual’s risk for developing CVD. By identifying high-risk patients, clinicians can recommend lifestyle modifications and medications to prevent the progression of CVDs, which can ultimately lead to terminal-stage heart failure if left untreated.

There are many different biomarkers that can be used to diagnose and predict the prognosis of CVD, each with varying levels of specificity and sensitivity. Because CVD is complex and multifactorial, it is often necessary to test the levels of multiple biomarkers to improve accuracy.

Cardiac biomarkers also play an important role in managing symptoms and predicting the risk of CVD complications in individuals who are being treated for chronic diseases other than CVD. Recently, the use of biomarkers has been particularly important in COVID-19 patients, as they can help clinicians stratify patients based on their risk for developing CVD and lower mortality rates. For example, levels of biomarkers of acute myocardial injury such as TnT, CK-MB, and NT-pro-BNP have been found to correlate with more severe symptoms of COVID-19. These biomarkers can also be used to guide therapeutic management based on drugs that prevent the activation of coagulation processes.

While there are some limitations to the use of biomarkers—such as the influence of nutritional status and medication on metabolite levels, and the need for validation of newly identified biomarkers—the advantages outweigh the limitations. Identifying a gold-standard cardiac biomarker will require the clinical evaluation of biomarkers in larger cohorts of patients within different populations.

In addition, identifying molecular partners involved in cardiovascular complications of COVID-19 is crucial for unveiling new prognostic predictors and therapeutic targets. While troponin levels have been found to be inconsistent as a marker of cardiac involvement in COVID-19 patients, dysregulations of circulating miRNAs have been associated with patient prognosis, multiorgan damage, and mortality. Therefore, identifying accessible and early biomarkers such as circulating miRNAs may also predict long-term cardiovascular consequences of COVID-19.

## Data Availability

Not applicable.
